# Probing Spin-Dependent Ballistic Charge Transport at Single-Nanometer Length Scales

**DOI:** 10.1021/acs.nanolett.3c03404

**Published:** 2023-12-14

**Authors:** Patrick Härtl, Markus Leisegang, Jens Kügel, Matthias Bode

**Affiliations:** † Physikalisches Institut, Experimentelle Physik II, Universität Würzburg, Am Hubland, 97074 Würzburg, Germany; ‡ Wilhelm Conrad Röntgen-Center for Complex Material Systems (RCCM), Universität Würzburg, Am Hubland, 97074 Würzburg, Germany

**Keywords:** scanning tunneling microscopy, molecular nanoprobe, spin-polarized, spin-momentum locking, spin transport, ballistic transport, molecular switch

## Abstract

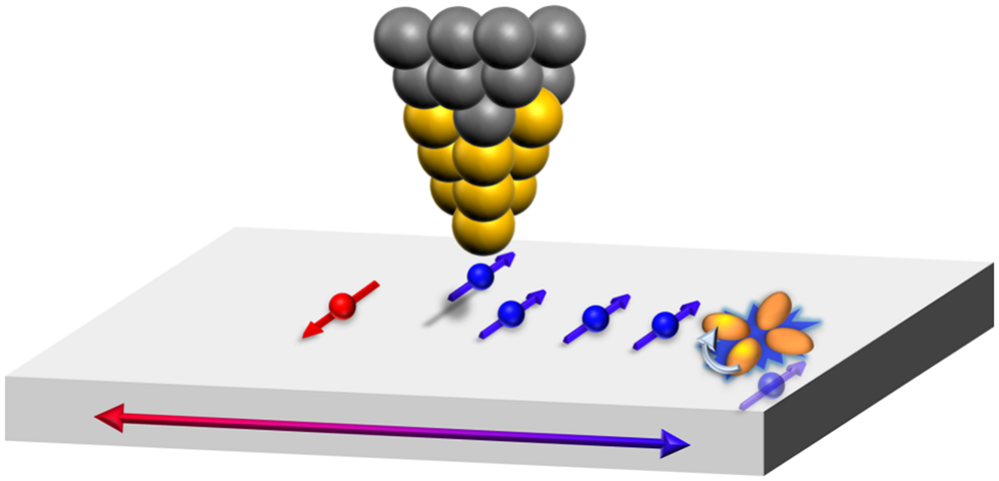

The coherent transport of charge and spin is a key requirement of future devices for quantum computing and communication. Scattering at defects or impurities may significantly reduce the coherence of quantum-mechanical states, thereby affecting the device functionality. While numerous methods exist to experimentally assess charge transport, the real-space detection of a material’s ballistic spin transport properties with nanometer resolution remains a challenge. Here we report on a novel approach that utilizes a combination of spin-polarized scanning tunneling microscopy (SP-STM) and the recently introduced molecular nanoprobe (MONA) technique. It relies on the local injection of spin-polarized charge carriers from a magnetic STM tip and their detection by a single surface-deposited phthalocyanine molecule via reversible electron-induced tautomerization events. Based on the particular electronic structure of the Rashba alloy BiAg_2_, which is governed by a spin-momentum-locked surface state, we prove that the current direction inverses upon tip magnetization reversal.

The progressing miniaturization of electronics components in integrated circuits has reached a point where single defects^1,2^ and the coherent superposition of quantum-mechanical states^1,3,4^ have to be considered. In fact, future technologies may fundamentally rely on nonlocal phase-coherent charge transfer processes, thereby enabling novel device concepts which materialize the enormous gain promised by quantum computation and communication, e.g., by utilizing Josephson tunneling junctions^3^ or zero-energy Majorana bound states.^5^ Particularly fascinating are strategies in which the conventional manipulation of charge is extended by the manipulation of the electron spin. For a long time, the concept of spintronics relied on the combination of nonmagnetic semiconductors with magnetic polarizers.^6,7^ The injection of spin-polarized charge carriers across material interfaces was a serious challenge in the past.^8^ In this context, formidable opportunities to overcome these limitations are concepts which utilize the spin-momentum locking^9−14^ of Rashba-split surface or interface states^15,16^ or topologically protected boundary states.^17−19^ In fact, the discovery of Aharonov–Bohm oscillations in topological insulators^20^ or the observation of Datta–Das oscillation in the ballistic intrinsic spin Hall effect^21^ clearly demonstrate that the coherent propagation of quantum-mechanical electronic states is a viable approach toward future spintronic devices.

In spite of the high expectations in the combination of spin-momentum locking and spintronics, our capabilities in detecting the spatial distribution of coherent spin currents are quite limited. The existence of edge channels has been demonstrated by imaging the current-induced magnetic fields in HgTe quantum wells by means of SQUID microscopy with micrometer resolution,^22^ but these data lack intrinsic spin sensitivity. Optical Kerr imaging is able to visualize spin transport in lateral ferromagnet/semiconductor structures,^23^ but the lateral resolution is limited by the wavelength of light. Shorter transport distances can be probed by lithographically prepared Hall bars, but the predefined electrode configuration cannot be changed and material damage may occur during processing.^24^ Nanoscopic resolution transport mapping, the detection of coherent transport along dimer rows of Ge(001),^25^ and spin transport measurements in pristine topological surface states with magnetic tips^26,27^ have been achieved by multiprobe STM setups operated in the scanning tunneling potentiometry mode,^28−32^ but this method is limited to intertip distances ≳30 nm.^33−38^

With the development of the molecular nanoprobe (MONA) technique, we are now able to achieve the detection of coherent ballistic charge transport on length scales down to the single nanometer limit. In this technique, charge carriers locally injected by an STM tip propagate across the surface and are detected by a single molecule via a reversible electron-induced switching process, such as a tautomerization.^39^ Charge transport in surface states,^40,41^ anisotropic transport on fcc(110) surfaces,^42^ and the damping and amplification by coherent superposition of quantum-mechanical waves in engineered atomic-scale structures has been experimentally demonstrated.^43^ In a recent study, the potential of MONA to measure transport in the Rashba-split surface state of the BiAg_2_ surface alloy has been proven.^44^ But so far, the spin component has been neglected in these measurements. In contrast, several publications have demonstrated that coherent phenomena in Rashba states can be probed on the atomic limit with STM and STS, revealing insights into the band structure and their spin-momentum locking. A well established method in STM is the d*I*/d*U*-mapping, where scattering of charge carriers at a given energy on step edges^45^ or even the confinement within artificially created atomic structures^46^ allow for an unprecedented insight into a possible scattering mechanism in Rashba-split surface states. By adding a magnetic tip to the system, the spin-polarized (SP)-STM technique can even locally map different spin domains, which might be the reason for slow carrier recombination in such a material.^47^

To go even beyond the mentioned studies and probe spin-dependent transport phenomena on the atomic limit, we combined the SP-STM method with the MONA technique to SP-MONA. In this work, we report on the development and application of spin-polarized (SP)-MONA. The capability of investigating the ballistic transport properties of spin-polarized charge carriers in real space on length scales of a few nanometers is exemplarily demonstrated by utilizing the spin-momentum-locked Rashba-split bands of the BiAg_2_ surface alloy.

As shown in Figure 1(a), BiAg_2_ features two downward dispersing surface states, an occupied *s*,*p*_*z*_-like band and a partially unoccupied *p*_*x*_,*p*_*y*_-derived band. Both bands exhibit a giant Rashba splitting of *E*_0_ – *E*_R_ ≈ 150 meV.^48−52^ The tunneling spectrum presented in Figure 1(b) shows two peaks that indicate the onset energies *E*_0_ of the Rashba-split surface states.

**Figure 1:**
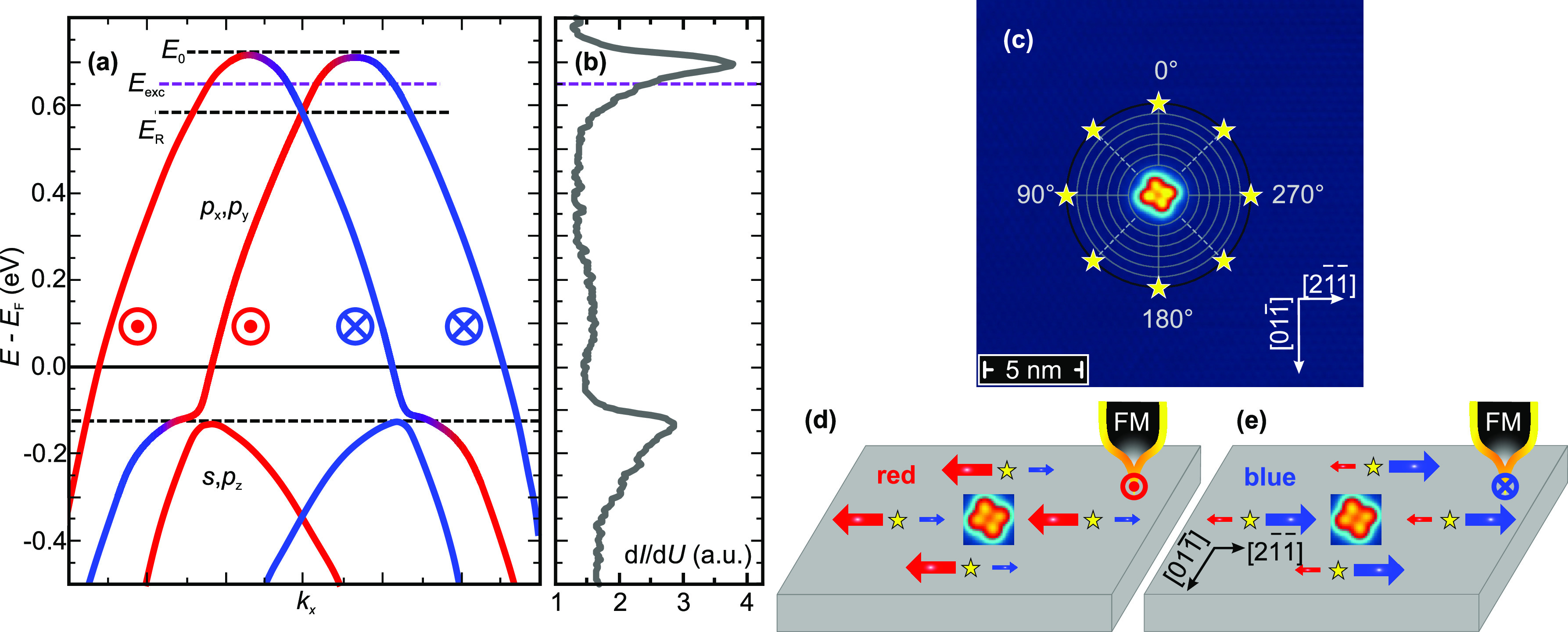
BiAg_2_ Rashba-split surface state. (a) Schematic representation of the Rashba-split bands where the spin of blue (red) bands points into (out of) the drawing plane. Adapted with permission from ref ^48^. Copyright 2013 by the American Physical Society. (b) d*I*/d*U* spectrum of the BiAg_2_ surface with peaks indicating the onsets of two downward dispersing Rashba-split surface states at *E*_1_ ≈ −130 meV and *E*_2_ ≈ 700 meV. (c) STM image of a single HPc molecule on BiAg_2_. For MONA measurements, charge carriers are injected at equiangular positions on a circle around the molecule, as marked by yellow stars. STM parameters: *U*_scan_ = 200 mV, *I*_scan_ = 100 pA. (d, e) Schematic drawings for the expected directional transport of a spin-polarized current injected by an SP tip into the unoccupied surface state at the four yellow stars. The color, length and thickness of the arrows represent the preferred direction as well as the expected strength of the spin-resolved currents.

The unoccupied *p*_*x*_,*p*_*y*_-derived bands exhibit an unconventional spin polarization,^45^ characterized by a reversal at the onset of the band, *E*_0_, as schematically represented by a transition from red to blue in Figure 1(a). This unusual Rashba splitting leads to a spin-dependent charge carrier propagation, which is generally given by the group velocity *v*^g^ = ∇_*k*_*E*,^53^ i.e., the derivative of the energy with respect to the crystal momentum. In the following, without limiting the generality of our considerations, we discuss states with *k*_*y*_ = 0. Inspection of Figure 1(a) reveals that electrons carrying a blue-colored spin (⊗) move with a negative *v*_⊗_^g^ = ∇_*k*_*E* < 0, whereas electrons with a red-colored spin (⊙) propagate in the opposite direction, *v*_⊙_^g^ = ∇_*k*_*E* > 0. As a consequence, we expect a striking real-space asymmetry of charge currents, with ⊙-electrons propagating to the left and ⊗-electrons moving to the right. We would like to emphasize that other magnetization directions might lead to group velocities that are no longer along *k*_*x*_.

To analyze this asymmetric propagation with the MONA technique, a single phthalocyanine (H_2_Pc) was placed on a defect-free area and subsequently deprotonated to HPc, the detector molecule; see Figure 1(c). Yellow stars mark the locations where charge carriers are injected from the STM tip (see Supporting Information Sections I and II for details). The charge-carrier-induced tautomerization of HPc serves as a measure for transport, presented as the normalized electron yield η in the following. Throughout the entire study, experiments will be performed at an energy *eU* = *E* – *E*_F_ = *E*_exc_ = 650 meV, marked by a purple dashed line in Figure 1(a). The energy is chosen such that it is well above the threshold *E*_tauto_ ≈ 408 meV for the molecular switch and, at the same time, below the band onset. The highest contribution to the detected electron yield is expected right at *E*_exc_, while the contribution of charge carriers with *E*_tauto_ < *E* < *E*_exc_ exponentially decreases with decreasing energy.^44^

As sketched in Figure 1(a) by the ⊙ and ⊗ symbols, the constant energy cut at *E*_exc_ is governed by spin-momentum locking, i.e., spins which are oriented perpendicular to the respective wave vector. Charge carriers with such an in-plane spin can be induced from a magnetically coated STM tip in the Rashba bands. The resulting asymmetry is expected to be strongest in the direction where the tip magnetization is collinear with the spin of the Rashba bands.^54,55^ For electrons with *k*_*y*_ = 0, this is the case for a tip magnetized along the in-plane  direction of BiAg_2_. As drawn in Figure 1(d), this should lead to the injection of red ⊙-electrons with a positive group velocity, resulting in a high (low) transport toward the molecule at α = 270° (α = 90°), i.e., an electron yield η_270_^red^ > η_90_^red^. Inverting the in-plane tip magnetization, see Figure 1(e), would result in the injection of ⊗-electrons with a negative group velocity. As a consequence, the preferred direction of charge transport would also invert, i.e., we expect η_90_^blue^ > η_270_^blue^. To quantify the spin polarization of charge transport when reversing the tip magnetization (*↑* or *↓*) by the application of a magnetic field μ_0_*H*, the asymmetry *A*_α_ of the electron yields η at a given angle α and can be calculated as *A*_α_ = (η_α_^↑^ – η_α_^↓^)/(η_α_^↑^ + η_α_^↓^). In contrast to an SP tip, the spin-averaged signal of a nonmagnetic tip should result in a vanishing asymmetry *A*_α_.

Figure 2 presents the results of measurements performed with (a) a nonmagnetic W tip and (b) a Gd-coated magnetic tip in polar coordinates. Each tip was treated in an external *↑*/*↓* magnetic field (red stars and blue circles, respectively) before the data were acquired in remanence (0 T). Charge carriers were injected with MONA parameters of *E*_exc_ = 650 meV, *t*_exc_ = 2.0 s, and *I*_exc_ = 1.0 nA at a distance of *d* = 4.0 nm from the molecule under four different angles. The data for a nonmagnetic W tip, Figure 2(a), show an electron yield η which, within error bars, is independent of the magnetic history of the tip. This can be quantified by an asymmetry |*A*_α_^NM^| < (2 ± 3)%. The small anisotropy of η between 0°/180° and 90°/270° results from the anisotropic coupling of the molecule to the substrate, as discussed in ref ^44^ and quantified in the Supporting Information Sections IV and V.

**Figure 2:**
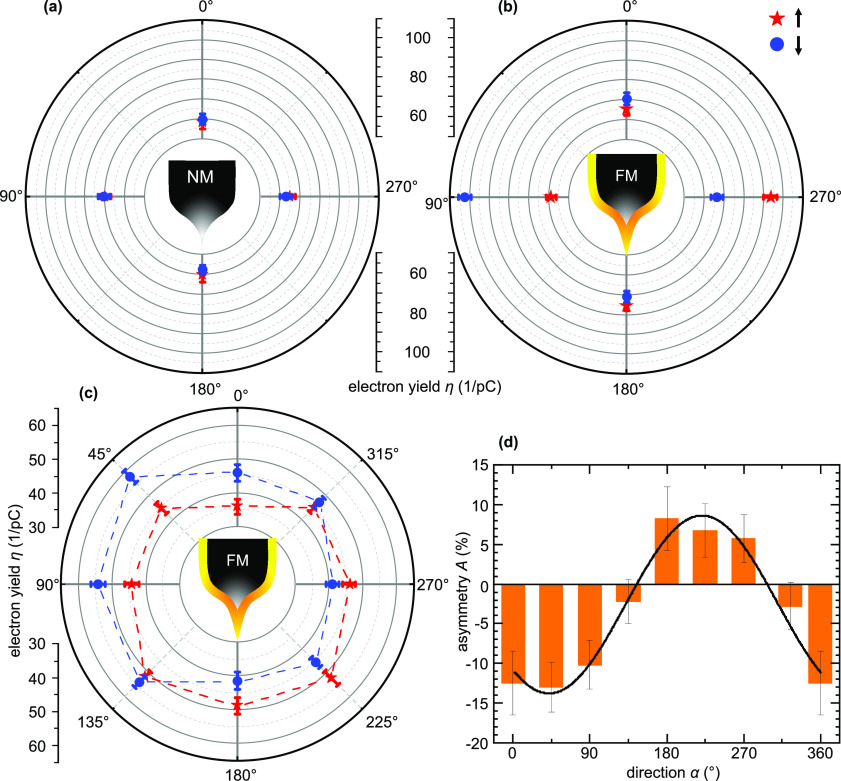
SP-MONA results. (a) MONA measurements with a nonmagnetic (NM) tungsten tip. The red/blue symbols in the polar plot represent the normalized electron yields η at four injection points taken after magnetization in an *↑*/*↓*-field and measured in remanence. (b) Same measurements as presented in (a) for a ferromagnetic (FM) Gd-coated tip. (c) Electron yield η taken at eight injection points with a different Gd tip in 45° steps for both field directions in remanence. The dashed lines serve as a guide to the eye. (d) Bar graph of the calculated asymmetry *A* between both field sweeps (orange bars) of (c), indicating a cosine behavior (fit, black line). MONA parameters: *E*_exc_ = 650 meV, *t*_exc_ = 2.0 s, *I*_exc_ = 1 nA (a, b), and *I*_exc_ = 1.5 nA (c).

In contrast, the data presented in Figure 2(b) for charge carriers injected from a magnetically coated Gd tip depend on the magnetic history, i.e., whether the tip was magnetized in a positive (*↑*) or negative (*↓*) field. While the *↑*/*↓* data points at 0° and 180° coincide within the error bars, resulting in low asymmetries *A*_0_^FM^ = (−4 ± 3)% and *A*_180_^FM^ = (3 ± 3)%, a significant deviation can be observed at 90° and 270°. The *↓*-tip results in a high (low) electron yield at 90° (270°), which inverts upon a *↑*-treatment. Quantitative analysis results in *A*_90_^FM^ = (−27 ± 3)% and *A*_270_^FM^ = (17 ± 3)%. These data are in line with our hypothesis in Figure 1(d, e).

Indeed, postcharacterization of the specific Gd-coated tip used for the experiments of Figure 2(b) on a test sample with Fe/W(110) monolayer islands confirms a significant in-plane polarization along the 0°–180° direction which can be inverted by an external field (see Supporting Information Section IX for details). Already at this point we can conclude that the absence of a significant asymmetry for a nonmagnetic tip in combination with the strong asymmetry observed for the magnetically Gd-coated tip proves that SP-MONA allows one to detect spin-dependent transport in the spin-momentum-locked Rashba-split surface state of the BiAg_2_ alloy.

To further substantiate this claim, we conducted MONA measurements at eight different angles (Δα = 45°) with a macroscopically different Gd-coated tip. Charge carriers were injected at a distance of *d* = 4.5 nm from the detector molecule. In Figure 2(c) the results measured in remanence after the tip was treated in an external *↑*/*↓* magnetic field are shown in a polar plot. Along the 135°–315° direction, the data points obtained with *↑* and *↓* magnetized tips coincide within the error bars, whereas a significant difference can be observed for the other six angles. The quantitative analysis reveals a cosine-like behavior of the asymmetry *A*, as presented in Figure 2(d), which can be fitted by *A*(α) = *O* + *b* cos(α – α_0_). Hereby, α_0_ = (40.4 ± 0.1)° represents the direction with the largest asymmetry, *A*_0_ = (−13.7 ± 0.1)%. We speculate that the offset *O* = (−2.5 ± 0.2)% is caused by an imperfect inversion of the tip magnetization during the field sweep, resulting in slightly different in-plane projections in remanence.

Given that a constant-energy cut of the spin-momentum-locked surface state reveals two concentric circles with tangential spins while the tip’s magnetization direction is not predetermined, the measured cosine-like behavior represents the projection of the tips in-plane magnetization onto these circles. Therefore, a vanishing asymmetry is obtained when the tip’s in-plane magnetization is along the tip–molecule axis, whereas the maxima of the asymmetry are observed when the injected spins are oriented orthogonal with respect to the tip–molecule axis.

The experimental data presented in our studies were obtained on perfect surfaces utilizing the well-known spin-momentum-locked electronic structure of the Rashba surface alloy BiAg_2_. An additional, unique capability of this technique lies in the analysis of atomically imperfect surfaces where charge and spin transport is affected by, e.g., the presence of vacancies, interstitials, domain boundaries, or adatoms.

As a first step toward measurements on atomic surface imperfections, we conducted SP-MONA experiments across a magnetic cluster, while a second setup with an additional molecule without any defect nearby serves as a reference. Both setups were probed with the same magnetic tip. The results, which are presented and discussed in detail in the Sections VI and VII of the Supporting Information, exhibit a significant change of charge carrier propagation across the cluster, affecting not only the absolute transport but also the degree of spin-polarization detected by the molecular probe. Unfortunately, the deposition of an unknown number of Gd-atoms from the tip upon gentle contact with the surface results in a cluster geometry and size that is beyond our control. Therefore, a conclusive interpretation of the influence of magnetic impurities on the ballistic spin-polarized transport remains an open challenge. Our results, however, encourage further measurements of more well-defined magnetic and nonmagnetic defects, to study the effect of atomic-scale impurities on spin-dependent ballistic transport.

Our study shows that SP-MONA is a unique experimental method which allows probing of ballistic charge transport properties at previously inaccessible length scales. As an STM-derived technique, the transport data can directly be correlated to topographic data, thereby allowing an assessment of how crystallographic imperfections at surfaces or interfaces affect spin transport. While the Rashba-split surface state of the BiAg_2_ surface provided an ideal testbed to demonstrate the general capabilities of SP-MONA, it is important to stress that SP-MONA is by no means restricted to materials with bands at or close to the Γ̅ point of the surface Brillouin zone, such as topological insulators (TIs).^20^ Since the propagation direction of ballistic charge carriers is determined by the group velocity, *v*^g^ = ∇_*k*_*E*, bands with qualitatively different slopes will always lead to opposite current directions. Therefore, also two-dimensional (2D) materials like graphene,^56^ bismuthene,^57^ or transition metal chalcogenides^58^ will be highly interesting materials for future experiments.

## Data Availability

The data that support the findings of this study are available from the corresponding authors upon reasonable request.

## Supporting Information Available

Experimental setup and sample preparation, MONA technique, rotational states of HPc on BiAg_2_, nonmagnetic tip, magnetic tip, MONA on a cluster, precharacterization of the magnetic tip on Gd/W(110), and postcharacterization of the magnetic tip on Fe/W(110) ML-islands (PDF)
