# Supplementary material for: Probing Spin-Dependent Ballistic Charge Transport at Single-Nanometer Length Scales
Source: Nano Lett. 2023 Dec 14;23(24):11608–13. doi: 10.1021/acs.nanolett.3c03404 (PMC10755752; doi:10.1021/acs.nanolett.3c03404)
Supplement: Supplementary file 1 — nl3c03404_si_001.pdf [file nl3c03404_si_001.pdf]

Supplementary Information for: "Probing Spin-Dependent Ballistic Charge Transport at Single-Nanometer Length Scales"

Patrick Härtl,† Markus Leisegang,*,† Jens Kügel,† and Matthias Bode†

†*Physikalisches Institut, Experimentelle Physik II, Universität Würzburg, Am Hubland, 97074 Würzburg, Germany*

‡*Wilhelm Conrad Röntgen-Center for Complex Material Systems (RCCM), Universität Würzburg, Am Hubland, 97074 Würzburg, Germany*

E-mail: markus.leisegang@uni-wuerzburg.de

I. Experimental setup and sample preparation

Experimental setup

The results were obtained in a two-chamber ultra-high vacuum (UHV) system (base pressure $p \leq 5 \times 10^{-11}$ mbar). STM measurements were carried out in the constant-current mode with a home-built low-temperature system with a base temperature of $T_{\mathrm{STM}} \approx 4.5\,\mathrm{K}$, with the bias voltage applied to the sample. A magnetic field oriented perpendicular to the surface plane with $|\mu_0 H| \leq 3\,\mathrm{T}$ can be generated by a superconducting split-coil magnet.

Sample preparation

Clean Ag(111) was prepared by cycles that consisted of 30 min Ar-ion sputtering at an energy of $E_{\mathrm{Ar}} = 500\,\mathrm{eV}$ and consecutive annealing at $T_{\mathrm{ann}} \approx 700\,\mathrm{K}$ for 20 min. In order to

achieve the well-ordered $BiAg_2$ alloy with the $(\sqrt{3} \times \sqrt{3})$Bi/Ag(111)$R30^\circ$ reconstruction, 1/3 of a pseudomorphic monolayer Bi was deposited onto the clean Ag(111) surface from a home-built Knudsen cell evaporator. During the deposition of Bi, the sample was held at elevated temperatures of $T_{\text{sample}} \approx 550\,\text{K}$. To reduce the defect density, the sample was afterwards held at $T_{\text{sample}} \approx 500\,\text{K}$ for one more minute.[1–4] H_2Pc molecules (Sigma-Aldrich) were deposited from a four-pocket Knudsen cell evaporator (Dodecon) onto the sample held at room temperature.[4]

Molecule manipulation

As reported previously,[4] H_2Pc molecules tend to adsorb at step edges or defects rather than on flat terraces of the $BiAg_2$ surfaces. Therefore, single molecules had to be moved to a defect-free surface area by means of STM manipulation. The manipulation was performed while scanning over the molecule and thereby dragging it. Typical tunneling parameters for this process were $U_{\text{bias}} \leq 20\,\text{mV}$ and $I_{\text{set}} > 5\,\text{nA}$. Eventually, the excitation barrier of the detector molecule was reduced by deprotonation of H_2Pc to HPc with a voltage pulse $U_{\text{bias}} \geq 2.5\,\text{V}$.

The overall measurement procedure

The overall measurement procedure contains eight steps: (i) In-situ preparation and pre-characterization of the magnetic tip on Gd(0001)/W(110) films; (ii) Sample exchange to $H_2Pc/BiAg_2$; (iii) Manipulation of H_2Pc molecules from step edges into defect-free surface areas; (iv) Application of an out-of-plane $\uparrow$ / $\downarrow$ magnetic field to align the tip; (v) MONA measurements in remanent field; (vi) Apply magnetic field in the opposite field direction to invert the tip polarization; (vii) MONA measurements in remanent field; (viii) Post-characterization of the magnetic tip on Fe/W(110) monolayer islands to verify the in-plane polarization and the inversion of the tip magnetization at the $\uparrow$ / $\downarrow$ magnetic field.

II. The MONA technique

The MONA technique allows the detection of ballistic charge carrier transport on the nanometer scale by charge carrier induced switching events of single molecules. This method has been established in our group, while the applicability has been proven in various publications.[4–10] The measurement setup consists of two probes: the STM tip which injects charge carriers into the surface which are detected by a single molecule, acting as second probe.

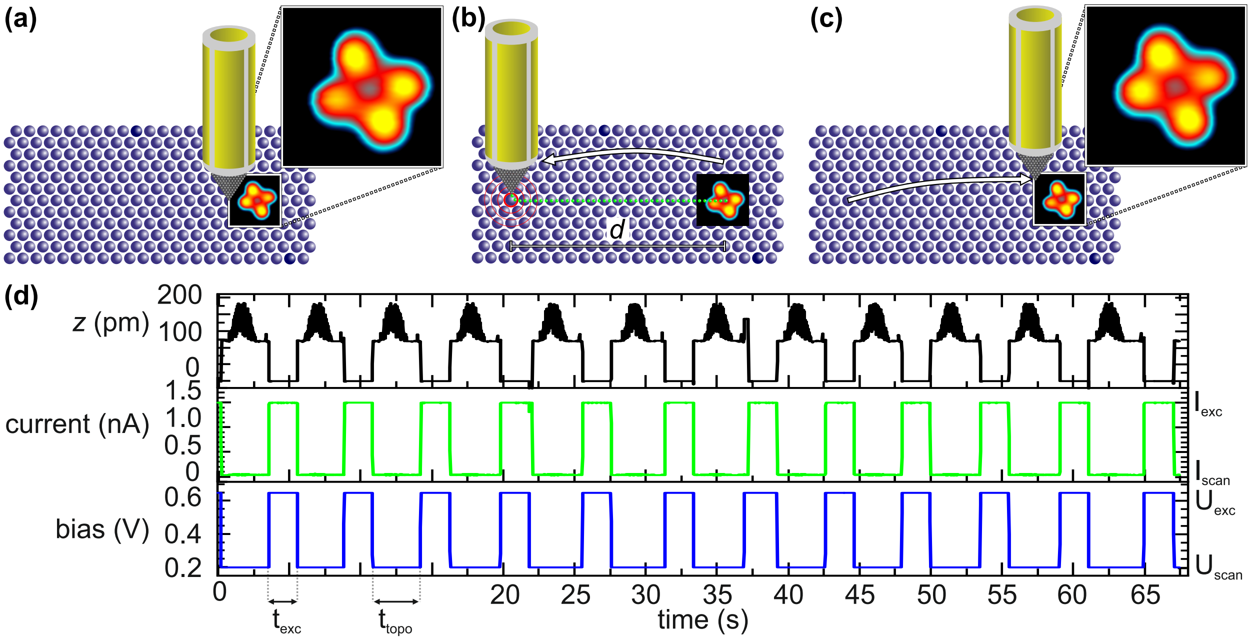

Figure 1: **The MONA technique.** (a) Topographic scan of an HPC molecule before a MONA measurement. (b) STM tip is moved to a distance d away from the molecule, where charge carriers (green dots) are injected at I_{exc}, U_{exc} for t_{exc} into the surface. (c) Tip is moved back to the molecule for a control topography scan of the HPC molecule indicating a tautomerization event compared to (a). (d) History for twelve injections with consecutive topography measurements of a MONA series. Top row – black: z–profile of the scheme; middle row – green: current with I_{exc} and I_{scan} during excitation and scanning; bottom row – blue: bias voltage U_{exc} and U_{scan} accordingly. Scan parameters for (a) and (c): $U_{\text{scan}} = 200\,\text{mV}$, $I_{\text{scan}} = 40\,\text{pA}$.

The general measurement principle is illustrated in Fig. 1:

a: Topographic scan before excitation at non-invasive parameters U_{scan}, I_{scan}.

b: Charge carriers are injected at a distance d from the molecule center for t_{exc} with I_{exc}

at U_{exc}.

c: Topographic scan after excitation at non-invasive parameters U_{scan}, I_{scan}.

The history in Fig. 1(d) visualizes the z profile (top – black), current (middle – green) as well as the bias voltage (blue – bottom) during twelve repetitions of the former introduced scheme. The non invasive parameters for topographic scans (high, fluctuating z–value) can be recognized by the low current ($I_{\mathrm{scan}} = 40\,\mathrm{pA}$) and bias values ($U_{\mathrm{scan}} = 200\,\mathrm{meV}$). The switch to excitation parameters ($U_{\mathrm{exc}}, I_{\mathrm{exc}}$) during injection is indicated by a jump in the z-height to low values. The injection and detection cycle is repeated up to 4000 times, taking into account the statistical nature of this procedure and to significantly reduce the standard deviation. As a measure, the electron yield η is extracted by dividing the counted tautomerization events by the number of injected charge carriers ($I_{\mathrm{exc}} \cdot t_{\mathrm{exc}}$).

III. Rotational states of HPc on $BiAg_2$

HPc adsorbed on $BiAg_2$ exhibits, next to the four tautomeric states, three rotational states. As shown in the topographic images of Fig. 2, three rotational states of molecules coexist, which are rotated by 120° towards each other and which will be labeled Rot A, Rot B, and Rot C hereafter. In each case the molecular arms are rotated by 45° from the substrates

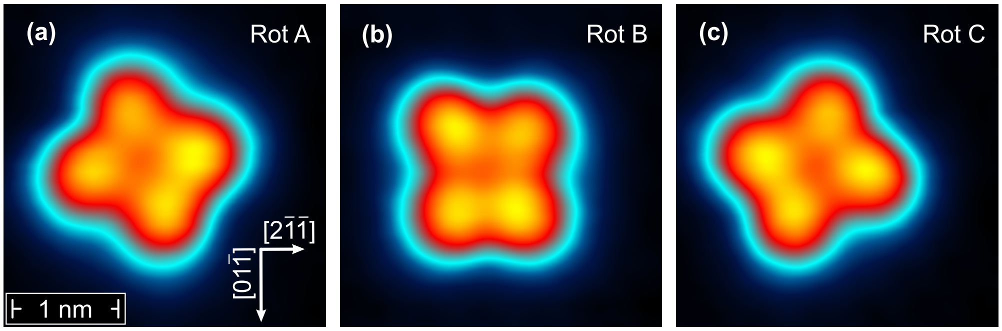

Figure 2: **The three rotational states of HPC on $BiAg_2$.** STM images of the states (a) Rot A, (b) Rot B, and (c) Rot C, which are rotated by 120° against each other. Scan parameters: $U_{\mathrm{scan}} = 100 \ldots 200\,\mathrm{meV}$, $I_{\mathrm{scan}} = 30 \ldots 40\,\mathrm{pA}$.

high-symmetry axes. A detailed study of the rotational behavior and the influence of the individual states on remote excitation has been reported in a recent study by us[4].

In this work we have shown that two distinct axes with different electron yields for tautomerization exist, resulting in a significant influence of the rotational state on the remote excitation. Moreover, the MONA measurements have revealed that these three states Rot A, Rot B, and Rot C are equally populated, indicating their equivalence. Within a MONA measurement, each data point includes several thousand single injection and detection events. Based on the statistical nature and the rather low probability for a rotation, however, which is by a factor of 30 lower as compared to a tautomerization[4], the absolute number of states Rot A, Rot B, and Rot C within an individual data set deviates. To account for this statistical variation and to correct the influence of an unequal state population, we weighted the experimentally determined tautomeric electron yield of all three rotational states by a factor of 1/3 which results in a realistic expectation value. In the following three sections, we will discuss the data presented in the main text with regard to the three rotational states. All data were recorded in remanence while the tip was pre-treated in an external out-of-plane oriented magnetic field of $\pm 2\,\mathrm{T}$. The asymmetry A_α of the electron yields η at a given angle α can be calculated as $A_\alpha = \left(\eta_\alpha^{+2\,\mathrm{T}} - \eta_\alpha^{-2\,\mathrm{T}}\right) / \left(\eta_\alpha^{+2\,\mathrm{T}} + \eta_\alpha^{-2\,\mathrm{T}}\right)$.

IV. Non-magnetic tip

Figure 3 shows spin-averaged MONA data recorded with a non-magnetic tungsten tip for all three rotations individually (a-c) as well as the total electron yield in (d) (non-weighted). MONA parameters are $E_{\mathrm{exc}} = 650\,\mathrm{meV}$, $t_{\mathrm{exc}} = 2.0\,\mathrm{s}$, $I_{\mathrm{exc}} = 1.0\,\mathrm{nA}$. The corresponding weighted data are shown in Fig. 2(a) of the main text. In all four plots, within the error bar the electron yield shows no correlation with the magnetic history, i.e., whether the data were recorded after a field sweep to positive or negative field.

However, a significant deviation between different angles can be found within one rota-

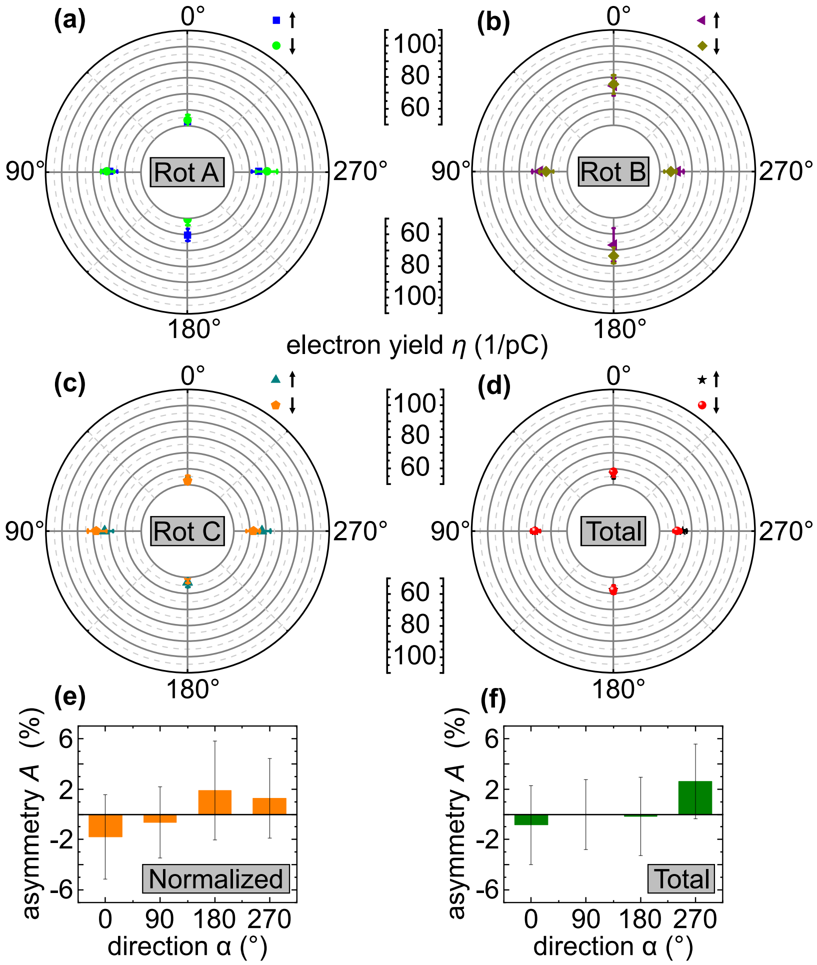

Figure 3: **MONA data for an unpolarized W-tip.** (a)-(c) Data for the spin-averaged measurement set [main text Fig. 2(a)] are plotted individually for each molecular rotation Rot A, Rot B and Rot C, respectively. All data have been measured in remanence (0 T) after exposing the tip to an external ↑ / ↓ magnetic field. (d) The non-weighted total electron yield, neglecting the influence of the rotations. (e) Weighted asymmetries of the data presented in Fig. 2(a) in the main text. (f) Asymmetries of the non-weighted total electron yield presented in (d).

tional state as well as for the same angle between the three rotational states. While the $0° - 180°$ axis shows a low electron yield for Rot A and Rot C, a high yield is observed along this axis for Rot B. This behavior inverts for the $90° - 270°$ axis. Now Rot A and Rot C have a high and Rot B has a low electron yield. As reported in detail in our recent publication, these observations can be explained by the combination of the four fold symmetric molecular cage with the six fold symmetric substrate[4]. Since two rotational states (Rot A and Rot B) exhibit the same behavior, they dominate the total electron yield (non-weighted) shown in Fig. 3(d) where the $90° - 270°$ axis has a higher yield as compared to the $0° - 180°$. For comparison, the calculated asymmetry A for the weighted and not-weighted averaged electron yield are presented in Fig. 3(e) and (d), respectively. In both cases, within the error bars no significant asymmetry can be found.

V. Magnetic tip

Figure 4 shows spin-polarized MONA data recorded with a magnetically Gd-coated tip for the three rotational states (a)-(c), as well as the total electron yield in (d) (non-weighted). The corresponding weighted data are shown in Fig. 2(b) of the main text. MONA parameters are $E_{\mathrm{exc}} = 650\,\mathrm{meV}$, $t_{\mathrm{exc}} = 2.0\,\mathrm{s}$, $I_{\mathrm{exc}} = 1.0\,\mathrm{nA}$ at a distance of $d = 4.0\,\mathrm{nm}$ from the molecule under four different angles. All four plots show a qualitative similar behavior. The two-fold symmetry of the electron yield with an inversion for Rot B is equivalent to what has been discussed for the unpolarized measurements. However, a significant deviation between the field sweeps can be observed. While the electron yields for both field sweeps ($\uparrow$ / $\downarrow$) are comparable along the $0° - 180°$ direction, a significant difference can be observed along the $90° - 270°$ axis for all three rotations as well as for the total sum. The calculated asymmetries

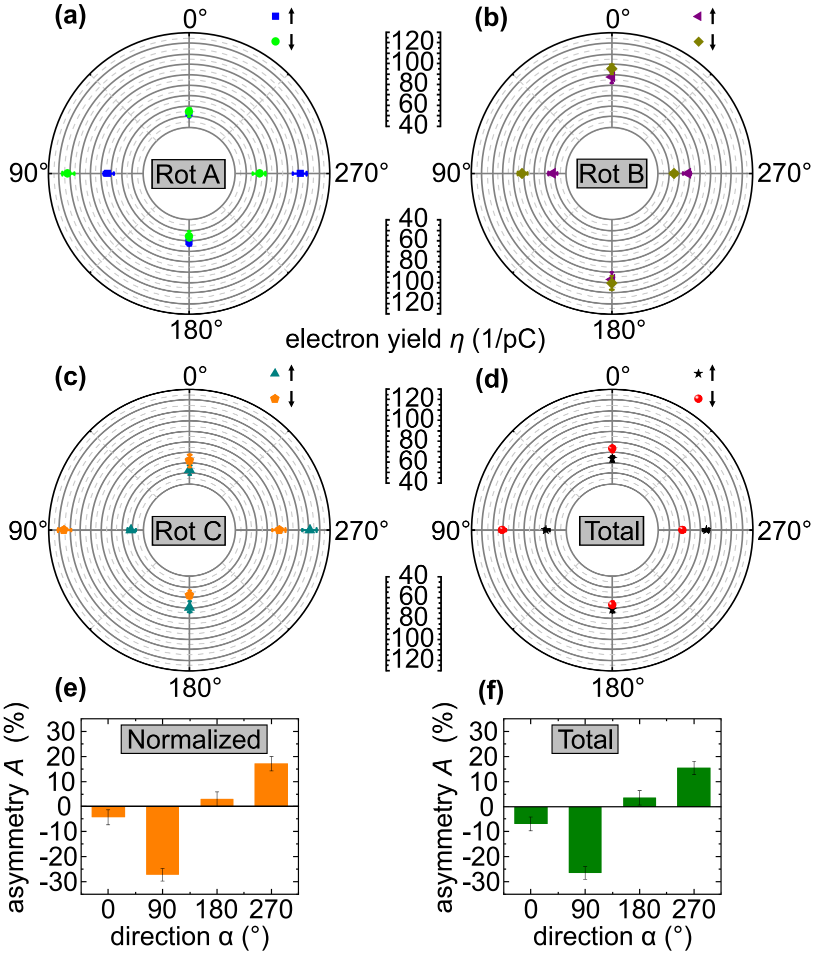

Figure 4: **MONA data measured with a magnetically coated Gd tip.** (a)-(c) Data for the spin-polarized measurement set [main text Fig. 2(b)] are plotted individually for each molecular rotation Rot A, Rot B and Rot C, respectively. All data have been measured in remanence (0 T) after exposing the tip to an external $\uparrow$ / $\downarrow$ magnetic field. (d) The non-weighted total electron yield, neglecting the influence of the rotations. (e) Weighted asymmetries for the data presented in Fig. 2(b) in the main text. (f) Asymmetries for the non-weighted total electron yield presented in (d).

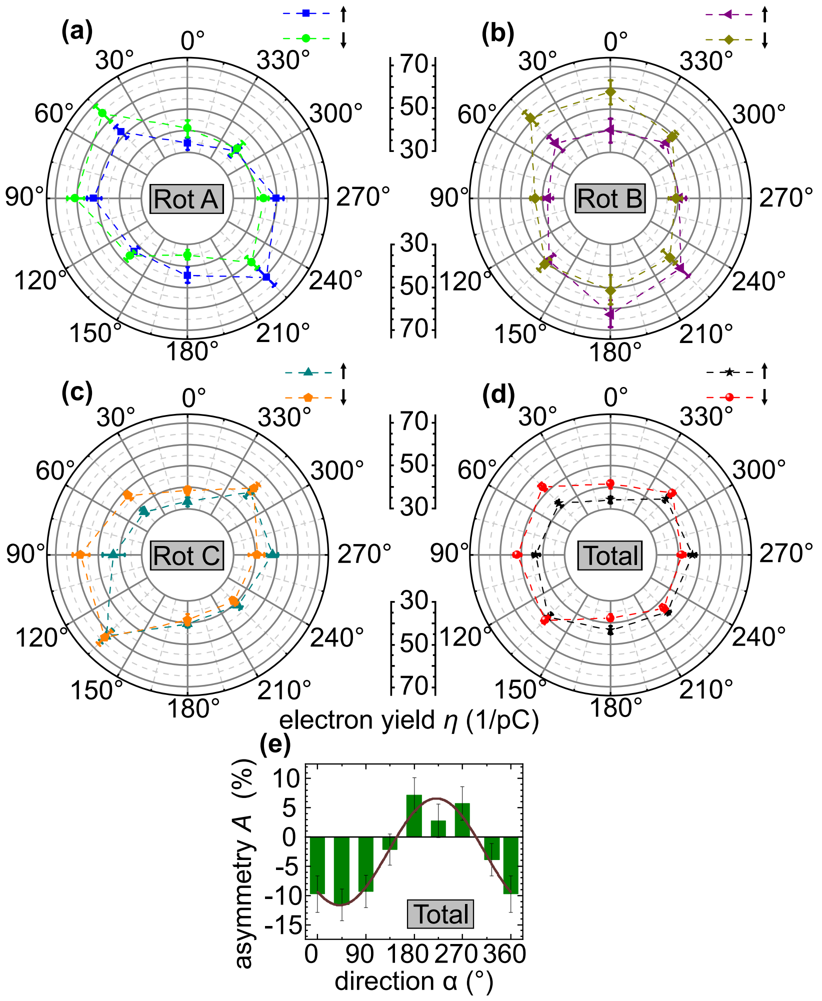

Figure 5: **High angular resolution spin-polarized MONA measurement.** (a)-(c) Data for the spin-polarized measurement set [main text Fig. 2(c)] are plotted individually for each molecular rotation Rot A, Rot B and Rot C, respectively. All data have been measured in remanence (0 T) after exposing the tip to an external ↑ / ↓ magnetic field. (d) The non-weighted total electron yield, neglecting the influence of the rotations. (e) Asymmetries for the non-weighted total electron yield presented in (d) with a cosine fit (brown line). The dashed lines in (a)-(d) serve as a guide to the eye only.

A for the weighted electron yields of the main text (Fig. 2(b)) as well as for the non-weighted total yield of Fig. 4 (d) are plotted in Fig. 4(e) and (f), respectively. Both plots reveal a similar behavior of A, while the absolute values slightly differ between the weighted and non-weighted electron yield η. Since all three rotations exhibit a significant asymmetry along the $90° - 270°$ axis, we can exclude a direct influence by the rotations.

Additional SP-MONA data, acquired with a macroscopically different magnetically Gd-coated tip, are presented in Fig. 5. MONA parameters are $E_{\text{exc}} = 650\,\text{meV}$, $t_{\text{exc}} = 2.0\,\text{s}$, $I_{\text{exc}} = 1.5\,\text{nA}$ at a distance of $d = 4.5\,\text{nm}$ from the molecule. These measurements provide a higher angular resolution with eight angles which are again plotted separately for all three rotations as well as the non-weighted total yield. The corresponding weighted data are shown in the main text Fig. 2(c). The dashed lines serve only as a guide to the eye. Due to the

higher resolution, a two-fold mirror symmetric electron yield can be observed for all three rotations. The plots show a rotation of roughly 120° towards each other, which is in line with the molecular rotations on the surface. However, the axis of high and low asymmetry A are always pointing along the $45° - 225°$ and $135° - 315°$ axes, respectively. These axes can also be identified in the non-weighted total electron yield in Fig. 5(d). The resulting asymmetry is shown in Fig. 5(e), where a cosine-like behavior can be fitted to the graph in accordance to the weighted data shown in the main text Fig. 2(c) and (d).

VI. SP-MONA on a cluster

To demonstrate the capability of SP-MONA, we conducted transport measurements across a magnetic cluster deposited on the $BiAg_2$ surface. The carefully designed setup is presented in the topographic STM image of Fig. 6(a). It consists of two HPc molecule which are placed at a distance of about 30 nm on a defect-free region of the $BiAg_2$ surface alloy. A Gd cluster was deliberately deposited from the STM tip at a distance of ≈ 4 nm from the left molecule. This molecule will allow for the spin-dependent detection of charge carrier transport injected at the four surrounding injection points (yellow stars, $d = 6.5$ nm) under the influence of the Gd cluster. In contrast, the right pristine HPc molecule is far from any defect or impurity and serves as a reference system.

Figure 6(b) and (c) depict the averaged and normalized electron yields at remanence for the two MONA setups in (a). While the plot in Fig. 6(c) with its pronounced asymmetry along the 90°–270° direction is in perfect agreement with the observation of spin-dependent charge carrier transport in a Rashba-split surface state reported in Figure 2 (b) in the main text, a strong influence of the cluster can be observed in Fig. 6(b). The overall electron yield across the cluster (data point at 90°) is significantly reduced, in-line with scattering events which would result in reduced ballistic transport between the injection point and the detector molecule. In contrast, the electron yield η measured for the other directions are

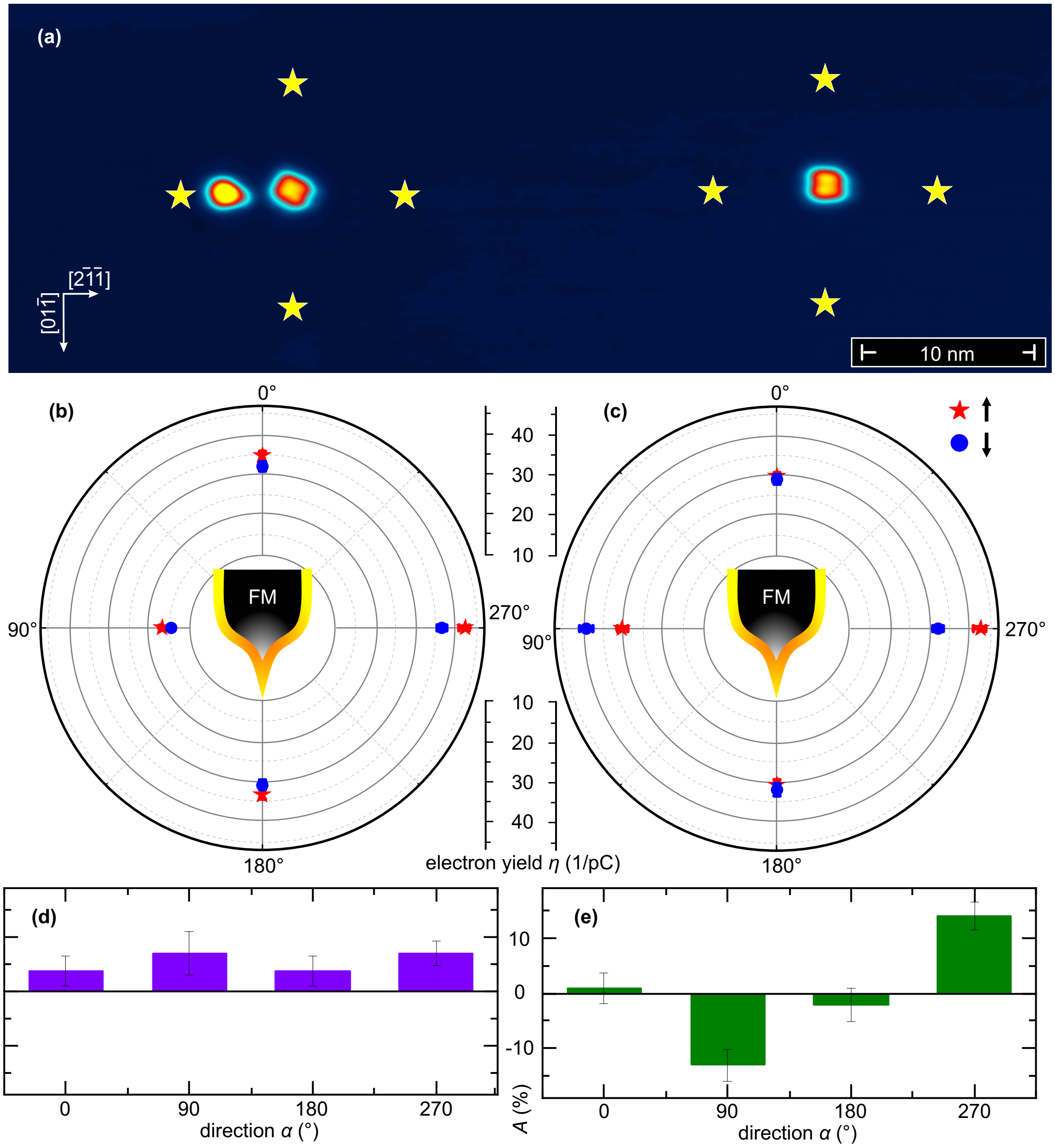

Figure 6: **Scattering at a Gd-cluster.** (a) Topographic STM image of two HPc molecules on a $BiAg_2$ surface, separated by approximately 30 nm along the $[2\bar{1}\bar{1}]$ direction. A Gd cluster which serves as a scattering center was deliberately deposited about 4 nm left of the left HPc molecule. The right molecule is used as a reference system without an additional scatterer. Yellow stars represent the injection points for SP-MONA measurements. STM parameters: $U_{\text{scan}} = 200\,\text{mV}$, $I_{\text{scan}} = 100\,\text{pA}$. (b) Polar plot of the electron yield η for the left molecule with the nearby Gd cluster, measured in remanence after the application of a $\uparrow/\downarrow$ field, see red/blue data points, respectively. (c) Same as (b) but for the right reference system. (d),(e) Asymmetries A of the tip magnetization direction-dependent electron yield of panels (b),(c). MONA parameters for both molecules: $t_{\text{exc}} = 2.5\,\text{s}$, $I_{\text{exc}} = 2.0\,\text{nA}$, $E_{\text{exc}} = 650\,\text{meV}$.

either equivalent or reveal a higher η as compared to the right setup. This may be caused by the constructive superposition of quantum-mechanical states, as previously observed in similar experiments.[7]

The calculated asymmetries allow for a discussion of possible spin-dependent effects. Indeed, the data of the right reference setup, see Fig. 6(e), are consistent with expected cosine-like behavior. In contrast, an overall positive and partially reduced asymmetry is observed under the influence of the Gd cluster, see Fig. 6(d). Since both measurements were conducted with the very same tip, the changes clearly result from the presence of the Gd cluster. Two possible influences need to be taken into account. On the one hand, the cluster acts as a scattering center. As mentioned before, this could cause either a reduction of ballistic transport based on elastic as well as inelastic processes, changing the propagation direction as well as reducing the charge carrier energy. Additionally, the interference of scattered charge carriers could lead to an even more complex situation. Unfortunately, a detailed analysis of scattering events with dI/dU mapping is beyond this work, since the wavelength $\lambda \approx 18\,\text{nm}$ at the injection energy $E_{\text{exc}} = 650\,\text{meV}$ is large as compared to the setup. On the other hand, the magnetic properties of the Gd cluster could cause spin-flip scattering events.

We would like to highlight that a thorough description of the magnetic properties of the cluster and therefore of the magnetic interaction with charge carriers is not possible. Since the size as well as shape of the cluster are unknown due to the deposition of Gd atoms from the tip, the properties as well as a reproducible experiment are beyond our control. However, the data, especially the reversal at $\alpha = 90^\circ$ from $A_{90} = (-14 \pm 3)\%$ for the clean surface to $A_{90} = (7 \pm 4)\%$ across the Gd cluster is quite surprising and encourage an interpretation based on possible magnetic properties. Since the cluster exhibits an apparent height $h_{\text{Gd}} = (173 \pm 5)\,\text{pm}$ and a diameter $d_{\text{Gd}} = (1.16 \pm 0.13)\,\text{nm}$, only a small amount of several atoms can be included herein. Additionally, Gd with its rather spherical charge distribution usually exhibits a relatively low magneto-crystalline anisotropy. Therefore, one could naively expect

that the cluster is superparamagnetic even at the measurement temperature of about 5 K.

However, as reported in Stern-Gerlach experiments on mass-selected Gd clusters, the magnetic behavior of rare-earth nanostructures is strongly size-dependent and most likely also depends on the precise atomic arrangement.[11] Furthermore, since the Gd cluster are deposited on a $BiAg_2$ surface in our experiments, the interaction with the high-Z substrate potentially increases the otherwise rather small magneto-crystalline anisotropy energy, possibly resulting in stable ferromagnetic order, i.e., a cluster magnetization which remanently follows magnetic field sweeps. A net magnetization might lead to spin-flip scattering events, resulting in the observed asymmetry pattern. However, these speculative considerations cannot be verified due to complex interplay of an unknown cluster's size, shape, and the interaction to the substrate. To gain deeper insights, comparative experiments with well-defined non-magnetic and magnetic clusters need to be conducted.

VII. SP-MONA on a cluster - Detailed Analysis

In this measurement, a Gd-cluster has been placed in the 90° direction as a magnetic impurity. Charge carriers were injected by a Gd-coated tip. MONA parameters are $E_{\mathrm{exc}} = 650\,\mathrm{meV}$, $t_{\mathrm{exc}} = 2.5\,\mathrm{s}$, and $I_{\mathrm{exc}} = 2.0\,\mathrm{nA}$ at a distance of $d = 6.5\,\mathrm{nm}$ from the molecule. The data are presented equivalently to the spin-averaged and spin-polarized data sets of Figs. 3 and 4, respectively. In the three plots of Fig. 7(a)-(c) we document the results obtained for all molecular rotation listed above. The averaged data of Fig. 7(d) clearly reveal the influence of the Gd cluster under 90°. The asymmetry shown in Fig. 7(e) clearly deviates from a cosine-like behavior, highlighting the strong influence of the cluster. The weighted data of this experimental run are shown in the main text Fig. 3(b) and (d).

An equivalent representation is given for a HPc without any defect nearby, acting as reference system for the measurements with a cluster. This setup has been probed on the very same surface with the same Gd-coated tip in a distance of around 30 nm to the setup

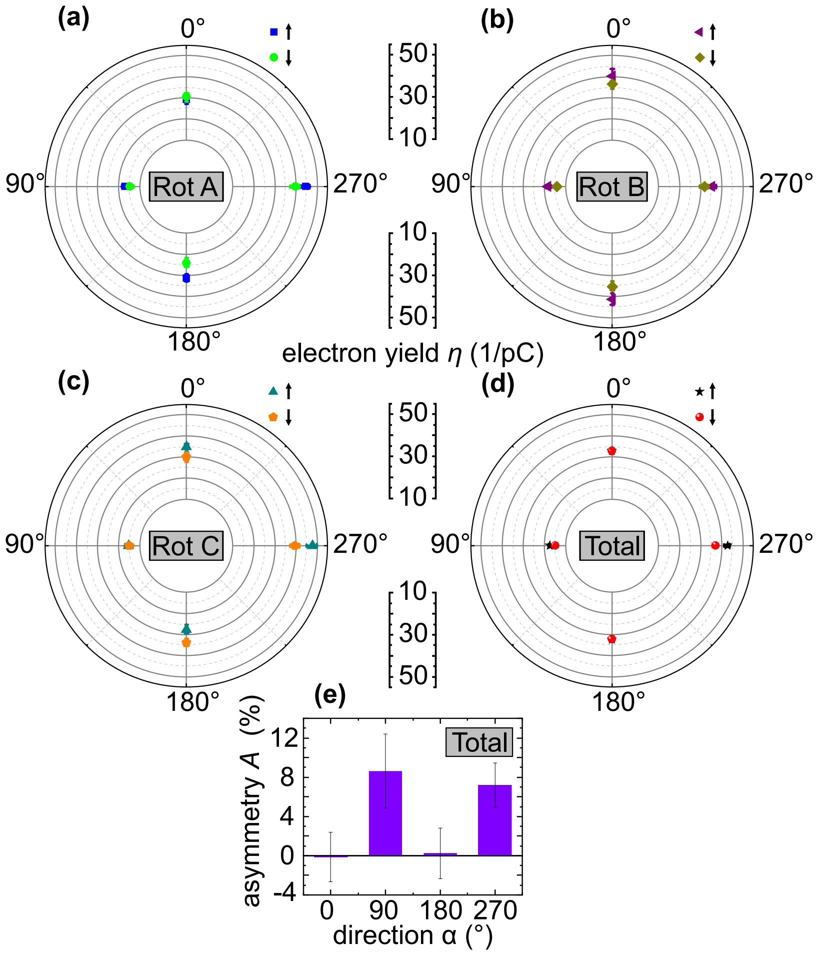

Figure 7: **SP-MONA data with a Gd-cluster.** (a)-(c) Data for the spin-polarized measurement set [main text Fig. 3(b)] are plotted individually for each molecular rotation Rot A, Rot B and Rot C, respectively. All data have been measured in remanence (0 T) after exposing the tip to an external ↑ / ↓ magnetic field. (d) The non-weighted total electron yield, neglecting the influence of the rotations. (e) Asymmetries for the non-weighted total electron yield presented in (d).

with cluster. The data in Fig. 8 show an equivalent cosine-like behavior (see asymmetry in Fig. 8(e)) as discussed for the SP-MONA data in Fig. 4 as well as the same signature originating from the molecular rotations.

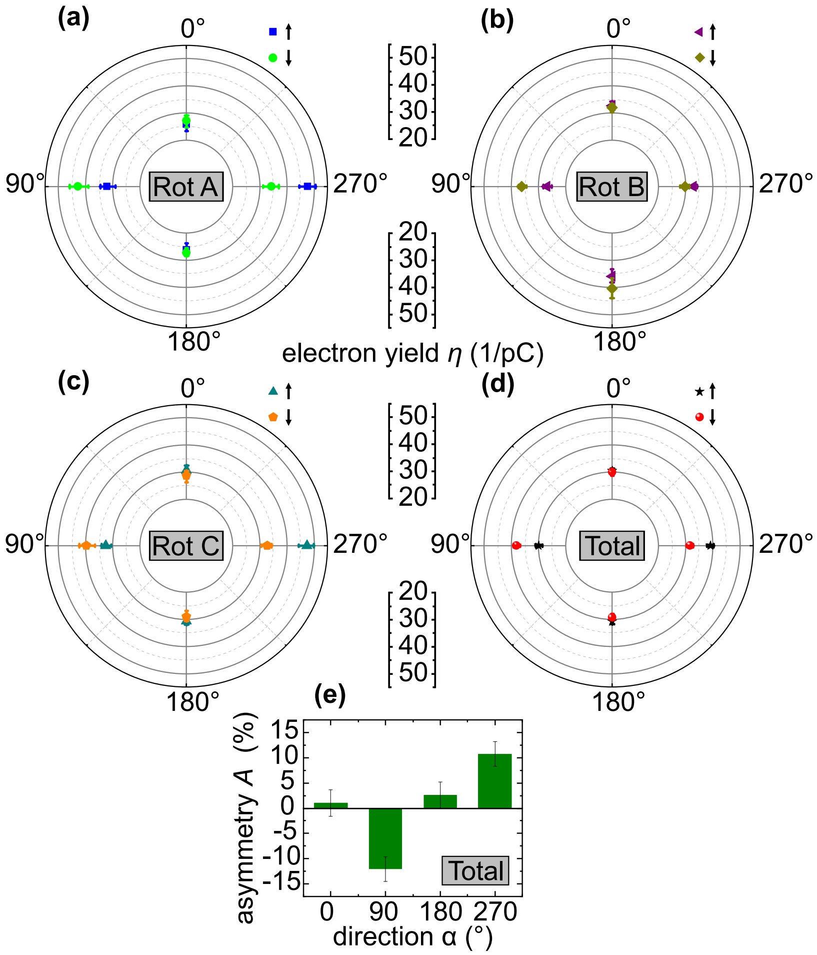

Figure 8: **Reference SP-MONA data without a Gd-cluster.** (a)-(c) Data for the spin-polarized measurement set [main text Fig. 3(c)] are plotted individually for each molecular rotation Rot A, Rot B and Rot C, respectively. All data have been measured in remanence (0 T) after exposing the tip to an external ↑ / ↓ magnetic field. (d) The non-weighted total electron yield, neglecting the influence of the rotations. (e) Asymmetries for the non-weighted total electron yield presented in (d).

VIII. Pre-characterization of the magnetic tip

In this section, the preparation and characterization of a magnetically Gd-coated tip is described. W tips are electro-chemically etched in a NaOH solution, carefully rinsed with distilled water, transferred into the ultra-high vacuum (UHV) system via a load lock, and then cleaned in several high-temperature flashes from potential contamination by e-beam heating.

To magnetize these initially non-magnetic tips, we utilize Gd(0001)/W(110) films which were investigated by us in a recent SP-STM study.[12] Fig 9(a) shows the topography of a 200 AL Gd film grown on a W(110) substrate. This film was deposited at a deposition rate of (19.8 ± 3.7) AL per minute onto the substrate held at room temperature. Subsequent annealing for five minutes at $T_{\text{anneal}} \geq 900\,\text{K}$ results in a very flat and highly ordered surface,

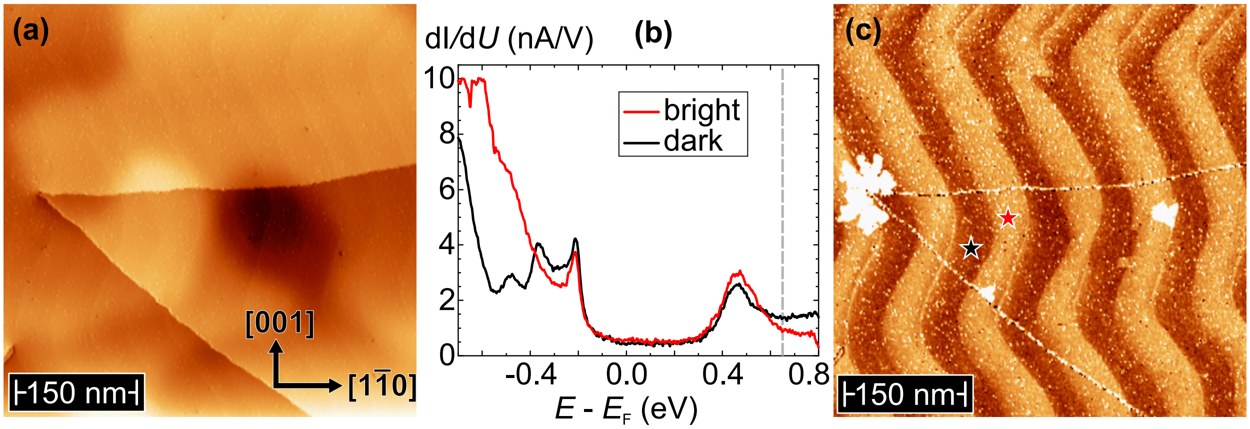

Figure 9: **Tip pre-characterization on Gd/W(110).** (a) Overview scan of a 200 AL Gd film epitaxially grown on a W(110) substrate. (b) dI/dU point spectra taken at the positions marked by a red/black star in (c), revealing a tip polarization at $E - E_\mathrm{F} = E_\mathrm{exc} = 650\,\mathrm{meV}$ (grey dashed line). (c) Magnetically sensitive dI/dU map of the film presented in (a). A zig-zag like domain pattern along the W[001] direction can be identified, comparable to the data already published by Härtl *et al.*[12] Scan parameters: $U_\mathrm{bias} = 650\,\mathrm{mV}$, $I_\mathrm{set} = 1\,\mathrm{nA}$.

see Ref. 12 for details.

The transfer of magnetic material (Gd) from this sample to the tip is achieved by dipping the tip a few nanometers into the Gd film or by gentle pulsing $U_\mathrm{bias} > 4\,\mathrm{V}$. To our experience both procedures may result in the attachment of a Gd cluster to the tip. To verify that the transfer resulted in a magnetically sensitive tip, we measured differential conductance dI/dU maps at $E - E_\mathrm{F} = E_\mathrm{exc} = 650\,\mathrm{meV}$. Only if the tip is indeed magnetically sensitive, a zig-zag pattern with two distinct contrast levels like the one shown in Fig. 9(c) appears, which is characteristic for the domain structure of Gd films in this thickness range.[12] At the points marked in the bright (red star) and the dark area (black star), dI/dU point spectra were taken at a frequency of $f_\mathrm{mod} = 5.309\,\mathrm{kHz}$ (well above the cutoff frequency of the feedback loop) and with a modulation of $U_\mathrm{bias} = 10\,\mathrm{mV}$. These spectra are plotted in Fig. 9(b). The significant difference between theses spectra evidences its suitability for spin-polarized experiments. Most important for the measurements presented in this contribution is the fact that we find a significant polarization at $E - E_\mathrm{F} = E_\mathrm{exc} = 650\,\mathrm{meV}$, marked by a grey dashed line in Fig. 9(b).

IX. Post-characterization of the magnetic tip

After successful SP-MONA measurements on $BiAg_2$, we verified the tip polarization by post-characterization on Fe/W(110) monolayer islands. This is an ideal test system, since the islands exhibit a well-defined in-plane magnetization along the $[\bar{1}\bar{1}0]$ direction of the W(110) substrate.[13] This direction is aligned with the $0° - 180°$ axis in our SP-MONA experiments. Fe was deposited onto the clean W(110) substrate from a rod with a diameter of 2 mm via an electron beam evaporator at a flux of 10 nA for 30 s deposition, leading to a coverage of ≈ 0.24 AL. During growth the substrate is held at a temperature $T_{\mathrm{sample}} \approx 400$ K. The resulting islands on the stepped substrate can be seen in the overview scan of Fig.10(a).

To verify that the tip is sensitive to the in-plane component of the magnetization at the energy used for our SP-MONA experiments, i.e., $E - E_{\mathrm{F}} = E_{\mathrm{exc}} = 650$ meV, we measured differential conductance $\mathrm{d}I/\mathrm{d}U$ maps at exactly this energy. The corresponding image in Fig. 10(c) shows islands with two different contrast levels, indicating a significant in-plane polarization of the tip after the SP-MONA measurements. At the positions marked with the red (bright island) star and the black (dark island) star, $\mathrm{d}I/\mathrm{d}U$ point spectra are taken. The resulting spectra are plotted in Fig. 10(b), confirming a significant spin polarization at $E - E_{\mathrm{F}} = E_{\mathrm{exc}} = 650$ meV (grey dashed line).

Subsequently, we verified that the in-plane component along the $[\bar{1}\bar{1}0]$ direction of the W(110) substrate can indeed be reversed successfully by the application of an external magnetic field. We exposed the tip to fields of -2 T (↓), $+2$ T (↑) and back to -2 T (↓). After each field ramp the magnetic state of the tip was investigated at remanence (0 T). Although the field is approximately oriented along the surface normal, i.e., out-of-plane, the $\mathrm{d}I/\mathrm{d}U$ images presented in Fig. 10(d)-(f) verify that the magnetization of the spin-sensitive tip used for the SP-MONA measurements of Fig. 3 in the main paper: (i) can be reversed with the available external magnetic fields, (ii) exhibits a significant spin polarization at the energy used in SP-MONA experiments ($E - E_{\mathrm{F}} = E_{\mathrm{exc}} = 650$ meV), and (iii) exhibits a significant in-plane component of magnetization such that it can produce spin-polarized transport in

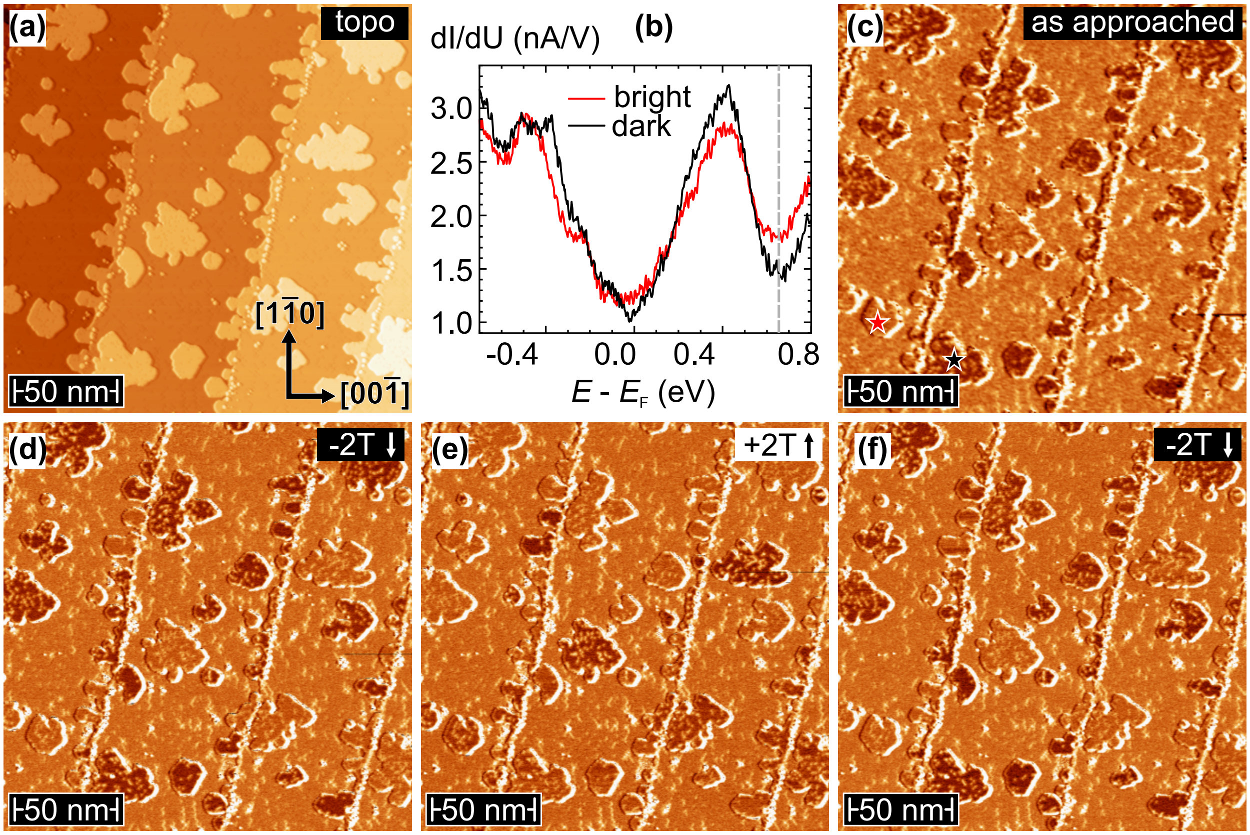

Figure 10: **Post-characterization of the magnetic tip on Fe/W(110) ML-islands.** (a) Topography of Fe monolayer islands on W(110) at a Fe coverage of $\approx 0.24\,\mathrm{AL}$. (b) $\mathrm{d}I/\mathrm{d}U$ point spectra measured on the Fe islands marked with a red/black star in (c), revealing a significant tip polarization at $E - E_\mathrm{F} = 650\,\mathrm{meV}$ (see grey hatched line) after the SP-MONA measurements. (c) $\mathrm{d}I/\mathrm{d}U$ map directly recorded after the SP-MONA measurement presented in Fig. 3 of the main text. The islands show two different contrast levels, indicating a significant in-plane polarization component of the magnetic STM tip. (d) $\mathrm{d}I/\mathrm{d}U$ map of the region shown in (c) after tip-treatment at $-2\,\mathrm{T}$ ($\downarrow$), measured in remanence. (e) $\mathrm{d}I/\mathrm{d}U$ map after a field ramp to $+2\,\mathrm{T}$ ($\uparrow$), measured in remanence. (f) $\mathrm{d}I/\mathrm{d}U$ map after a field ramp back to $-2\,\mathrm{T}$ ($\downarrow$), where the islands again inverted their contrast. STM parameters: $U_\mathrm{bias} = 650\,\mathrm{mV}$, $I_\mathrm{set} = 1\,\mathrm{nA}$.

the spin–momentum-locked Rashba states.

We can only speculate how and why the out-of-plane field is capable of reliably reversing the in-plane component of the tip magnetization. One option would be that the tip's easy magnetization axis is canted, i.e., in remanence it simultaneously exhibits an in-plane and an out-of-plane component of the magnetization. Furthermore, the magnetic field produced by superconducting coils is certainly not oriented perfectly along the surface normal but

somewhat canted. It may be possible that this field canting supports the reliable switching of the in-plane component. Yet, we would like to emphasize that the results presented in Fig. 10 unambiguously proof that a significant in-plane component exists and that it can reliably be reversed.

References

(1) Ast, C. R.; Henk, J.; Ernst, A.; Moreschini, L.; Falub, M. C.; Pacilé, D.; Bruno, P.; Kern, K.; Grioni, M. Giant Spin Splitting through Surface Alloying. *Phys. Rev. Lett.* **2007**, *98*, 186807.

(2) El-Kareh, L.; Sessi, P.; Bathon, T.; Bode, M. Quantum Interference Mapping of Rashba-Split Bloch States in Bi/Ag(111). *Phys. Rev. Lett.* **2013**, *110*, 176803.

(3) El-Kareh, L.; Bihlmayer, G.; Buchter, A.; Bentmann, H.; Blügel, S.; Reinert, F.; Bode, M. A combined experimental and theoretical study of Rashba-split surface states on the Pb/Ag(111) surface. *New J. Phys.* **2014**, *16*, 045017.

(4) Leisegang, M.; Böhme, M.; Maiberger, D.; Härtl, P.; Kügel, J.; Bode, M. Electron-Induced Switching Processes of Phthalocyanine Molecules on $(\sqrt{3} \times \sqrt{3})$ Bi/Ag(111)$R30°$: Tautomerization Accompanied by Rotation. *J. Phys. Chem. C* **2023**, *127*.

(5) Kügel, J.; Leisegang, M.; Böhme, M.; Krönlein, A.; Sixta, A.; Bode, M. Remote Single-Molecule Switching: Identification and Nanoengineering of Hot Electron-Induced Tautomerization. *Nano Lett.* **2017**, *17*, 5106–5112.

(6) Kügel, J.; Leisegang, M.; Bode, M. Imprinting Directionality into Proton Transfer Reactions of an Achiral Molecule. *ACS Nano* **2018**, *12*, 8733–8738.

(7) Leisegang, M.; Kügel, J.; Klein, L.; Bode, M. Analyzing the Wave Nature of Hot Electrons with a Molecular Nanoprobe. *Nano Lett.* **2018**, *18*, 2165–2171.

(8) Kügel, J.; Zenger, T.; Leisegang, M.; Bode, M. On the Impact of Geometrical Factors on Hot Electron-Induced Tautomerization. *J. Phys. Chem. C* **2019**, *123*, 17056–17061.

(9) Leisegang, M.; Zenger, T.; Bode, M.; Kügel, J. Guiding a Proton-Controlled Directionality in a Single Molecule. *J. Phys. Chem. C* **2020**, *124*, 10727–10732.

(10) Leisegang, M.; Schindhelm, R.; Kügel, J.; Bode, M. Anisotropic Ballistic Transport Revealed by Molecular Nanoprobe Experiments. *Phys. Rev. Lett.* **2021**, *126*, 146601.

(11) Douglass, D. C.; Cox, A. J.; Bucher, J. P.; Bloomfield, L. A. Magnetic properties of free cobalt and gadolinium clusters. *Phys. Rev. B* **1993**, *47*, 12874.

(12) Härtl, P.; Leisegang, M.; Bode, M. Magnetic domain structure of epitaxial Gd films grown on W(110). *Phys. Rev. B* **2022**, *105*, 174431.

(13) Krause, S.; Berbil-Bautista, L.; Herzog, G.; Bode, M.; Wiesendanger, R. Current-Induced Magnetization Switching with a Spin-Polarized Scanning Tunneling Microscope. *Science* **2007**, *317*, 1537–1540.
